# Supplementary material for: Effects of selection stringency on the outcomes of directed evolution
Source: PLoS One. 2024 Oct 14;19(10):e0311438. doi: 10.1371/journal.pone.0311438 (PMC11472920; doi:10.1371/journal.pone.0311438)
Supplement: S1 Appendix — (PDF) [file pone.0311438.s001.pdf]

SI Appendix for "Effects of selection stringency on the outcomes of directed evolution"

DFE properties under the two-parent model

The mean and standard deviation of an exponential DFE is the inverse of its rate parameter λ. In the two-parent model, this quantity $\beta = 1/\lambda$ is a random variable where $\lambda \sim \text{Exponential}(\alpha)$. Performing a transformation of variables, we have that

$$f_\beta(\beta) = \left|\frac{d\lambda}{d\beta}\right| f_\lambda(1/\beta) = \frac{\alpha}{\beta^2} e^{-\alpha/\beta}.$$

It follows that the distribution function is $F_\beta(x) = e^{-\alpha/x}$ and the quantile function is $Q_\beta(p) = \alpha/\log(1/p)$. The interquartile range is then

$$Q_\beta(3/4) - Q_\beta(1/4) = \frac{\alpha \log 3}{\log \frac{16}{9} \log 2} \approx 2.8\alpha.$$

The mode of the distribution is $\alpha/2$, the β at which $f'_\beta(\beta) = \alpha e^{-\alpha/\beta}(\alpha - 2\beta)/\beta^4 = 0$.

Mutant fitness under the two-parent model

In the main text, we analyze statistics of the expected log maximum mutant fitness. For ease of notation, let us abbreviate λ_{low} as λ_{L}, λ_{high} as λ_{H}, and n_{low} as n_{L}. The density of $M = \max\{x_1, \ldots, x_n\}$, the fitness of the fittest mutant, can be expressed as

$$p(M = m) = \int_0^\infty \int_0^\infty p(M = m|\lambda_{\text{L}}, \lambda_{\text{H}}) p(\lambda_{\text{L}}) p(\lambda_{\text{H}}) d\lambda_{\text{L}} d\lambda_{\text{H}}.$$

There are two ways this fittest mutant can arise: as a mutant of the more-fit variant in the previous round, or as a mutant of the less-fit variant in the previous round. Decomposing in this way, we have that

$$\begin{aligned} p(M = m|\lambda_{\text{L}}, \lambda_{\text{H}}) = {} & n_{\text{L}} p(x_{\text{L}} = m) p(x_{\text{L}} < m)^{n_{\text{L}}-1} p(x_{\text{H}} < m)^{n-n_{\text{L}}} \\ & + (n - n_{\text{L}}) p(x_{\text{H}} = m) p(x_{\text{H}} < m)^{n-n_{\text{L}}-1} p(x_{\text{L}} < m)^{n_{\text{L}}}, \end{aligned}$$

where x_{L} is the fitness of an arbitrary mutant of the less-fit variant, with DFE parameter λ_{L}, and x_{H} is the fitness of an arbitrary mutant of the more-fit variant with DFE parameter λ_{H}.

According to the two-parent model, $(x_{\text{L}} + \Delta x) \sim \text{Exponential}(\lambda_{\text{L}})$, $x_{\text{H}} \sim \text{Exponential}(\lambda_{\text{H}})$, and $\lambda_{\text{L}}, \lambda_{\text{H}} \overset{\text{i.i.d.}}{\sim} \text{Exponential}(\alpha)$. Thus,

$$\begin{aligned} p(M = m) = \alpha^2 \int_0^\infty \int_0^\infty & [n_{\text{L}} \lambda_{\text{L}} e^{-\lambda_{\text{L}}(m+\Delta x)} (1 - e^{-\lambda_{\text{L}}(m+\Delta x)})^{n_{\text{L}}-1} (1 - e^{-\lambda_{\text{H}} m})^{n-n_{\text{L}}} \\ & + (n - n_{\text{L}}) \lambda_{\text{H}} e^{-\lambda_{\text{H}} m} (1 - e^{-\lambda_{\text{H}} m})^{n-n_{\text{L}}-1} (1 - e^{-\lambda_{\text{L}}(m+\Delta x)})^{n_{\text{L}}}] \\ & e^{-\alpha(\lambda_{\text{L}}+\lambda_{\text{H}})} d\lambda_{\text{L}} d\lambda_{\text{H}}. \end{aligned}$$

Using Mathematica, we solve the integral $\text{E}(\log M) = \int_0^\infty p(M = m) \log(m) dm$ setting $n_{\text{L}} = 0$ and $n_{\text{L}} = n/2$ for various even n, and find the intersection.

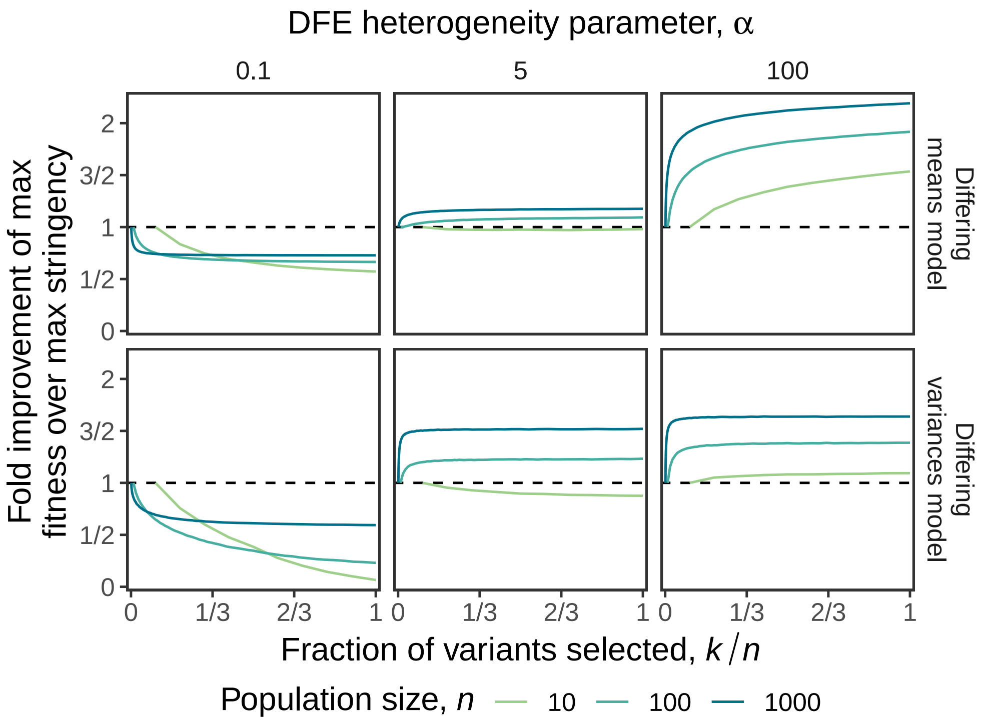

Supplementary Figure 1. The immediate effect of selection stringency on fitness in alternative k-parent models. As in Fig 3B, under two alternative models. In both models, $\lambda_i \overset{\text{i.i.d.}}{\sim} \text{Exponential}(1/\alpha)$, but in the first, the DFE of the ith parent is $\text{Normal}\left(\sqrt{\lambda_i}, 1\right)$, while in the second it is $\text{Normal}(0, \lambda_i)$.

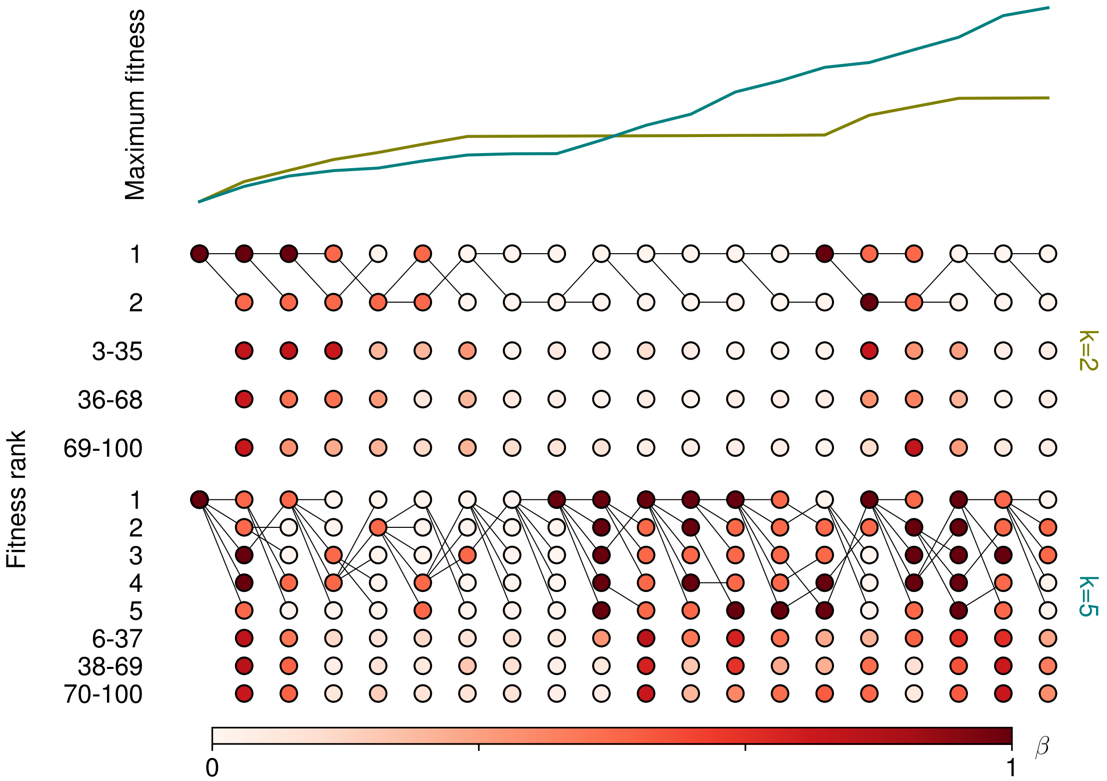

Supplementary Figure 2. Longer-term dynamics of fitness with respect to selection stringency when DFE favorability can be recovered. Example evolutions as in Fig 4A at two selection stringencies, assuming $p = d = 1/2$ and a probability $1/20$ of recovering to $\beta = 1$.
